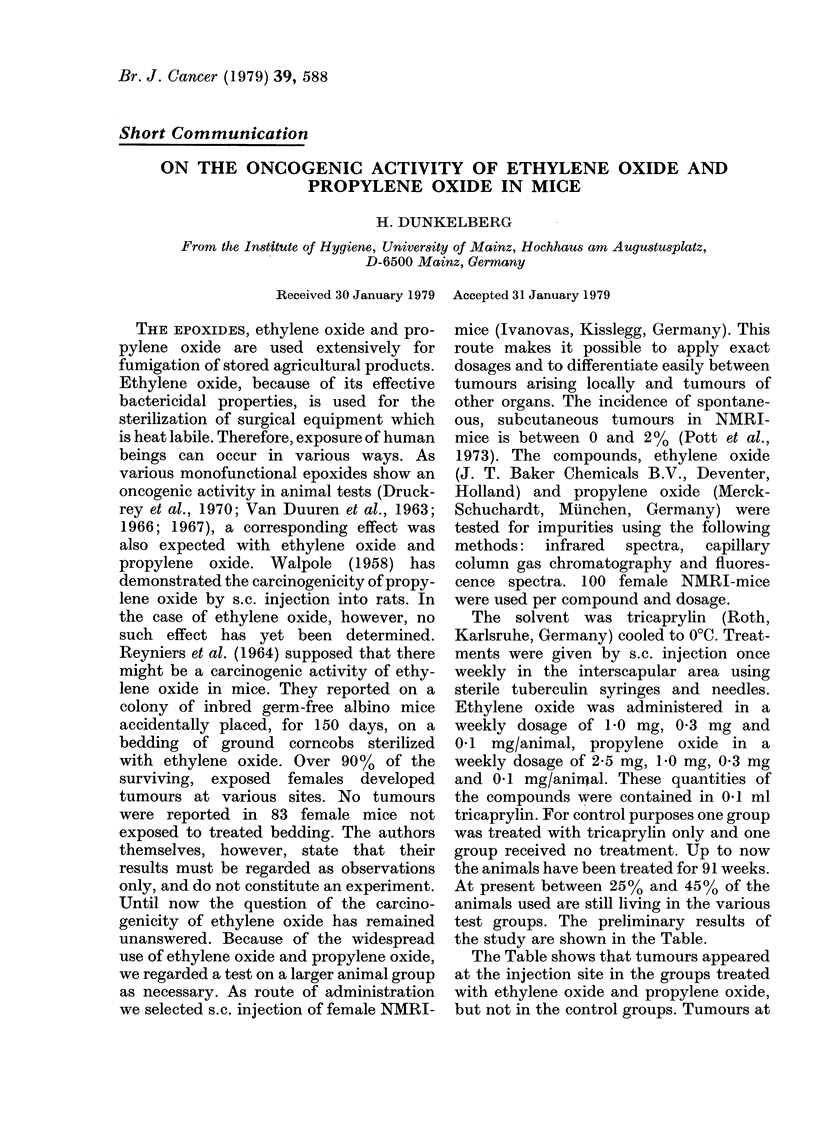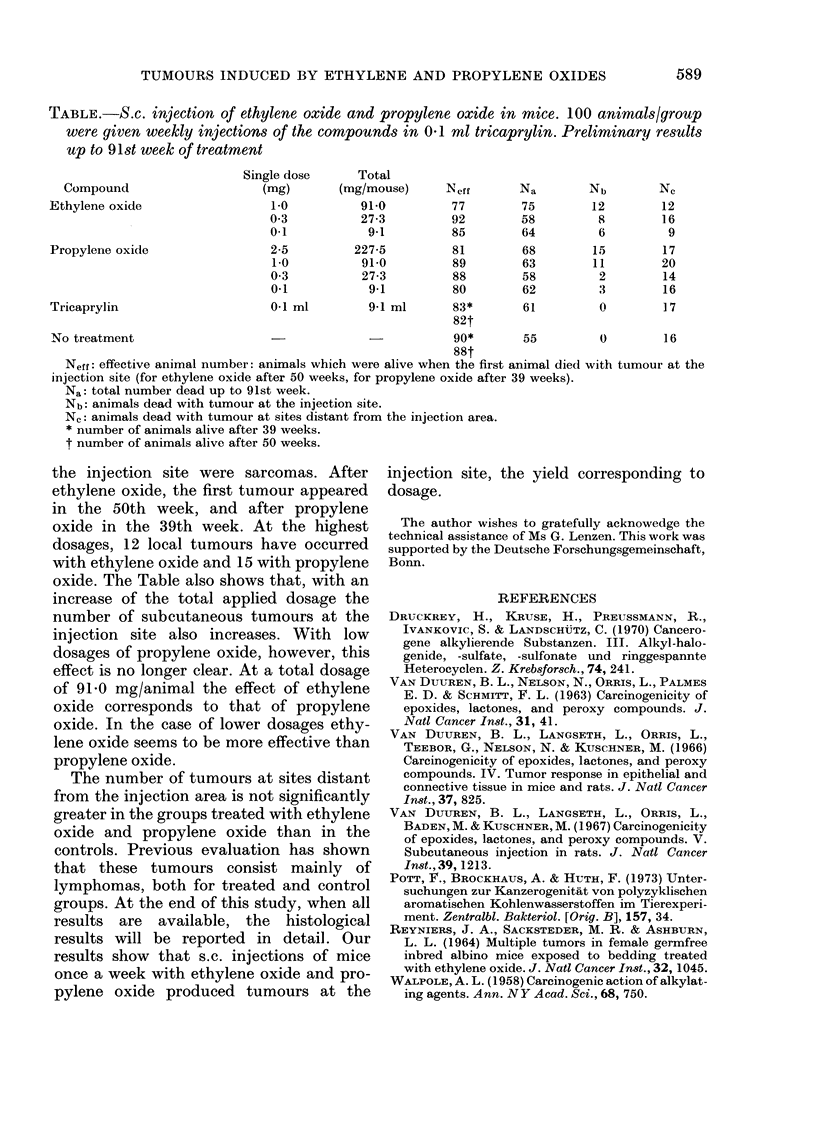# On the oncogenic activity of ethylene oxide and propylene oxide in mice.

**DOI:** 10.1038/bjc.1979.106

**Published:** 1979-05

**Authors:** H. Dunkelberg


*Br. J. Cancer* (1979) **39**, 588

***Short Communication***

### ON THE ONCOGENIC ACTIVITY OF ETHYLENE OXIDE AND PROPYLENE OXIDE IN MICE

H. DUNKELBERG

*From the Institute of Hygiene, University of Mainz, Hochhaus am Augustusplatz, D-6500 Mainz, Germany*

Received 30 January 1979 Accepted 31 January 1979

THE EPOXIDES, ethylene oxide and propylene oxide are used extensively for fumigation of stored agricultural products. Ethylene oxide, because of its effective bactericidal properties, is used for the sterilization of surgical equipment which is heat labile. Therefore, exposure of human beings can occur in various ways. As various monofunctional epoxides show an oncogenic activity in animal tests (Druckrey *et al.*, 1970; Van Duuren *et al.*, 1963; 1966; 1967), a corresponding effect was also expected with ethylene oxide and propylene oxide. Walpole (1958) has demonstrated the carcinogenicity of propylene oxide by s.c. injection into rats. In the case of ethylene oxide, however, no such effect has yet been determined. Reyniers *et al.* (1964) supposed that there might be a carcinogenic activity of ethylene oxide in mice. They reported on a colony of inbred germ-free albino mice accidentally placed, for 150 days, on a bedding of ground corncobs sterilized with ethylene oxide. Over 90% of the surviving, exposed females developed tumours at various sites. No tumours were reported in 83 female mice not exposed to treated bedding. The authors themselves, however, state that their results must be regarded as observations only, and do not constitute an experiment. Until now the question of the carcinogenicity of ethylene oxide has remained unanswered. Because of the widespread use of ethylene oxide and propylene oxide, we regarded a test on a larger animal group as necessary. As route of administration we selected s.c. injection of female NMRI-mice (Ivanovas, Kisslegg, Germany). This route makes it possible to apply exact dosages and to differentiate easily between tumours arising locally and tumours of other organs. The incidence of spontaneous, subcutaneous tumours in NMRI-mice is between 0 and 2% (Pott *et al.*, 1973). The compounds, ethylene oxide (J. T. Baker Chemicals B.V., Deventer, Holland) and propylene oxide (Merck-Schuchardt, München, Germany) were tested for impurities using the following methods: infrared spectra, capillary column gas chromatography and fluorescence spectra. 100 female NMRI-mice were used per compound and dosage.

The solvent was tricaprylin (Roth, Karlsruhe, Germany) cooled to 0°C. Treatments were given by s.c. injection once weekly in the interscapular area using sterile tuberculin syringes and needles. Ethylene oxide was administered in a weekly dosage of 1·0 mg, 0·3 mg and 0·1 mg/animal, propylene oxide in a weekly dosage of 2·5 mg, 1·0 mg, 0·3 mg and 0·1 mg/animal. These quantities of the compounds were contained in 0·1 ml tricaprylin. For control purposes one group was treated with tricaprylin only and one group received no treatment. Up to now the animals have been treated for 91 weeks. At present between 25% and 45% of the animals used are still living in the various test groups. The preliminary results of the study are shown in the Table.

The Table shows that tumours appeared at the injection site in the groups treated with ethylene oxide and propylene oxide, but not in the control groups. Tumours at

TABLE.—*S.c. injection of ethylene oxide and propylene oxide in mice.* 100 *animals/group were given weekly injections of the compounds in* 0·1 *ml tricaprylin. Preliminary results up to* 91*st week of treatment*

| Compound | Single dose (mg) | Total (mg/mouse) | $N_{eff}$ | $N_a$ | $N_b$ | $N_c$ |
|---|---|---|---|---|---|---|
| Ethylene oxide | 1·0 | 91·0 | 77 | 75 | 12 | 12 |
| | 0·3 | 27·3 | 92 | 58 | 8 | 16 |
| | 0·1 | 9·1 | 85 | 64 | 6 | 9 |
| Propylene oxide | 2·5 | 227·5 | 81 | 68 | 15 | 17 |
| | 1·0 | 91·0 | 89 | 63 | 11 | 20 |
| | 0·3 | 27·3 | 88 | 58 | 2 | 14 |
| | 0·1 | 9·1 | 80 | 62 | 3 | 16 |
| Tricaprylin | 0·1 ml | 9·1 ml | 83* 82† | 61 | 0 | 17 |
| No treatment | — | — | 90* 88† | 55 | 0 | 16 |

$N_{eff}$: effective animal number: animals which were alive when the first animal died with tumour at the injection site (for ethylene oxide after 50 weeks, for propylene oxide after 39 weeks).
$N_a$: total number dead up to 91st week.
$N_b$: animals dead with tumour at the injection site.
$N_c$: animals dead with tumour at sites distant from the injection area.
* number of animals alive after 39 weeks.
† number of animals alive after 50 weeks.

the injection site were sarcomas. After ethylene oxide, the first tumour appeared in the 50th week, and after propylene oxide in the 39th week. At the highest dosages, 12 local tumours have occurred with ethylene oxide and 15 with propylene oxide. The Table also shows that, with an increase of the total applied dosage the number of subcutaneous tumours at the injection site also increases. With low dosages of propylene oxide, however, this effect is no longer clear. At a total dosage of 91·0 mg/animal the effect of ethylene oxide corresponds to that of propylene oxide. In the case of lower dosages ethylene oxide seems to be more effective than propylene oxide.

The number of tumours at sites distant from the injection area is not significantly greater in the groups treated with ethylene oxide and propylene oxide than in the controls. Previous evaluation has shown that these tumours consist mainly of lymphomas, both for treated and control groups. At the end of this study, when all results are available, the histological results will be reported in detail. Our results show that s.c. injections of mice once a week with ethylene oxide and propylene oxide produced tumours at the injection site, the yield corresponding to dosage.

The author wishes to gratefully acknowedge the technical assistance of Ms G. Lenzen. This work was supported by the Deutsche Forschungsgemeinschaft, Bonn.

### REFERENCES

DRUCKREY, H., KRUSE, H., PREUSSMANN, R., IVANKOVIC, S. & LANDSCHÜTZ, C. (1970) Cancerogene alkylierende Substanzen. III. Alkyl-halogenide, -sulfate, -sulfonate und ringgespannte Heterocyclen. *Z. Krebsforsch.*, **74**, 241.

VAN DUUREN, B. L., NELSON, N., ORRIS, L., PALMES E. D. & SCHMITT, F. L. (1963) Carcinogenicity of epoxides, lactones, and peroxy compounds. *J. Natl Cancer Inst.*, **31**, 41.

VAN DUUREN, B. L., LANGSETH, L., ORRIS, L., TEEBOR, G., NELSON, N. & KUSCHNER, M. (1966) Carcinogenicity of epoxides, lactones, and peroxy compounds. IV. Tumor response in epithelial and connective tissue in mice and rats. *J. Natl Cancer Inst.*, **37**, 825.

VAN DUUREN, B. L., LANGSETH, L., ORRIS, L., BADEN, M. & KUSCHNER, M. (1967) Carcinogenicity of epoxides, lactones, and peroxy compounds. V. Subcutaneous injection in rats. *J. Natl Cancer Inst.*, **39**, 1213.

POTT, F., BROCKHAUS, A. & HUTH, F. (1973) Untersuchungen zur Kanzerogenität von polyzyklischen aromatischen Kohlenwasserstoffen im Tierexperiment. *Zentralbl. Bakteriol.* [*Orig. B*], **157**, 34.

REYNIERS, J. A., SACKSTEDER, M. R. & ASHBURN, L. L. (1964) Multiple tumors in female germfree inbred albino mice exposed to bedding treated with ethylene oxide. *J. Natl Cancer Inst.*, **32**, 1045.

WALPOLE, A. L. (1958) Carcinogenic action of alkylating agents. *Ann. NY Acad. Sci.*, **68**, 750.